# Effect of shading on physiological attributes and comparative transcriptome analysis of *Camellia sinensis* cultivar reveals tolerance mechanisms to low temperatures

**DOI:** 10.3389/fpls.2023.1114988

**Published:** 2023-02-02

**Authors:** Shah Zaman, Jiazhi Shen, Shuangshuang Wang, Dapeng Song, Hui Wang, Shibo Ding, Xu Pang, Mengqi Wang, Irfan Ali Sabir, Yu Wang, Zhaotang Ding

**Affiliations:** ^1^ Tea Research Institute, Shandong Academy of Agricultural Sciences, Jinan, China; ^2^ Tea Research Institute, Rizhao Academy of Agricultural Sciences, Rizhao, China; ^3^ School of Agriculture and Biology, Shanghai Jiao Tong University, Shanghai, China; ^4^ Tea Research Institute, Qingdao Agricultural University, Qingdao, China

**Keywords:** shading, tea, physiological attributes, transcriptome analysis, low temperature, signal transductions

## Abstract

Tea is a vital beverage crop all over the world, including in China. Low temperatures restrict its growth, development, and terrestrial distribution, and cold event variability worsens cold damage. However, the physiological and molecular mechanisms of *Camellia sinensis* under shade in winter remain unclear. In our study, tea leaves were utilized for physiological attributes and transcriptome analysis in November and December in three shading groups and no-shade control plants. When compared to the no-shade control plants, the shading group protected tea leaves from cold damage, increased photochemical efficiency (Fv/Fm) and soil plant analysis development (SPAD), and sustained chlorophyll *a*, chlorophyll *b*, chlorophyll, and carotenoid contents by physiological mean. Then, transcriptome analysis revealed 20,807 differentially expressed genes (DEGs) and transcription factors (TFs) in November and December. A comparative study of transcriptome resulted in 3,523 DEGs and many TFs under SD0% *vs.* SD30%, SD0% *vs.* SD60%, and SD0% *vs.* SD75% of shading in November and December. Statistically, 114 DEGs were downregulated and 72 were upregulated under SD0% *vs.* SD30%. SD0% *vs.* SD60% resulted in 154 DEGs, with 60 downregulated and 94 upregulated. Similarly, there were 505 DEGs of which 244 were downregulated and 263 were upregulated under SD0% *vs.* SD75% of shading throughout November. However, 279 DEGs were downregulated and 105 were upregulated under SD0% *vs.* SD30%. SD0% *vs.* SD60% resulted in 296 DEGs, with 172 downregulated and 124 upregulated. Finally, 2,173 DEGs were regulated in December, with 1,428 downregulated and 745 upregulated under SD0% *vs.* SD75%. These indicate that the number of downregulated DEGs in December was higher than the number of upregulated DEGs in November during low temperatures. Gene Ontology (GO) and Kyoto Encyclopedia of Genes and Genomes (KEGG) analyses of differentially expressed genes were highly regulated in the photosynthesis, plant hormone signal transduction, and mitogen-activated protein kinase (MAPK) signaling pathways. However, qRT-PCR and RNA-seq relative expression of photosynthetic (DEGs) *Lhcb2* in both November and December, plant hormone (DEGs) *BRI1* and *JAZ* in November and *IAA* and *ERF1* in December, and key DEGs of MAPK signal transduction *FLS2*, *CHIB*, and *MPK4* in November and *RBOH*, *MKK4_5*, and *MEKK1* in December in three shading groups and no-shade control plants responded to tea cold tolerance. The enhanced expression of light-harvesting photosystem I gene *Lhca5*, light-harvesting photosystem II gene *Lhcb2*, and mitogen-activated protein kinases *MEKK1* and *MPK4/6* enhance the cold-tolerance mechanism of *C. sinensis*. These comprehensive transcriptomic findings are significant for furthering our understanding of the genes and underlying regulatory mechanisms of shade-mediated low-temperature stress tolerance in horticultural crops.

## 1 Introduction

Tea *Camellia sinensis* is a beverage crop and a species of evergreen shrub or small tree with leaves, first discovered in China in 2737 B.C. The tea tree thrives in 10°C–30°C warm, humid climates (zone 8 climate or warmer). Due to climatic changes, tea plants undergo environmental and abiotic stresses such as cold, chilling, and hot and low temperatures, damaging plant development, quality, and yield (Zaman et al., 2022). Tea plants are susceptible to cold injury, especially during harsh winters (Li et al., 2019). Tea leaves are substantially harmed by cold stress, resulting in cellular disruption, decreased photosynthetic capability and chlorophyll formation, and other physiological changes that contribute to irregular growth and limit the tea plants’ healthy development to survive in harsh environments (Chen et al., 2018). To offset the loss of tea yield and production during winters, innovative climatic and resilient agricultural practices are needed to alleviate abiotic stresses and harmful effects of low temperatures and reduce cold damage in tea plants (Zaman et al., 2022).

The temperature of the environment has a vital role in plant growth and development. It regulates photosynthesis (food production) and metabolism (food utilization). Each plant species has a temperature range in which it grows best based on its origin (e.g., tropical or temperate). The temperature has an enormous impact on plant resilience, fall color, and senescence (leaf fall). The capability of a plant to tolerate the average minimum temperature of a region without damage or death is determined by its hardiness. Even cold-hardy plants are vulnerable to the devastating effects of a rapid temperature drop to freezing (Li et al., 2019). Plants experience chilling and freezing as low-temperature stresses. Chilling temperatures (0°C–15°C) vary by plant kind and tolerance. Air temperature and wind speed affect chilling temperatures. At temperatures below (0°C), most of the plants initiate ice formation in cellular compartments and begin to freeze (Thomashow, 2010), which disrupts membrane permeability and the growth and development of horticultural plants. The gradual shifts in photoperiod and temperature throughout the year are detected by perennial plants such as tea trees to predict the upcoming season. These plants can shift from active growth to dormant phases and from frost sensitivity to cold tolerance in response to seasonal cues, including day length and temperature fluctuation. However, the seasonal cold adaptation of woody perennials is caused mainly by short autumn days, further enhanced by low temperatures (Aslam et al., 2022). Overwintering plants’ morphological and physiological attributes coordinate with the climate’s cycles, ultimately leading to their survival in low, moderate, and icy environments (Sakai and Larcher, 2012). During low temperatures, plants develop a mechanism known as cold acclimation (Zhang et al., 2019). This cold acclimation is an induced adaptive process that increases cold tolerance and is accomplished by exposing the plant to numerous low temperatures depending on the plant’s adaptation to specific environments. Cold acclimation triggers a general response to cold stress. It can help avoid this cold damage, and the mechanisms of cold acclimation have been investigated incredibly well in forage legumes, cereal crops, and model plants like *Arabidopsis thaliana* (Baier et al., 2019). Tea plants may reduce the cold damage and sustain the photosystem systemic reaction of cold acclimation during cold stress (Li et al., 2019). Many significant crops, including rice, corn, soybean, potato, cotton, and tomato, are chilling sensitive and incapable of cold acclimation.

In contrast, other crops, such as oats, are chilling-tolerant but freezing-susceptible. However, barley, wheat, and rye are well suited to freezing conditions (Fu et al., 2011). Low temperatures alter the expression of thousands of differentially expressed genes (DEGs) and transcription factors, leading to metabolic rearrangement, physiological adaptation, and biochemical changes. Plant hormone changes are associated with multiple biological processes and complex regulatory networks (Thomashow, 1999). Changes in lipid composition, membrane fluidity, and structural organization occur in plants exposed to cold weather. Photosynthesis converts light energy into ATP and NADPH by chloroplast thylakoid membrane components. Low temperatures reduce the metabolic sink capacity for photosynthates; hence, photochemical rates must adjust. Low temperatures affect photosynthesis and carbon gain, determining plant productivity, growth, and distribution. Cold-tolerant species may adapt to low temperatures. Changes in energy absorption and photochemical transformation *via* energy partitioning, as well as contemporaneous changes in chloroplast carbon metabolism, allocation, and partitioning, are mechanisms for overcoming the restrictions of short-term and long-term exposure to low temperatures. Thus, the mechanisms involved in photosynthetic acclimation to low temperatures arise from thylakoid membrane system alterations that affect photosynthetic electron transport. The genes and transcription factors that trigger the signals in the photosynthesis pathway regulate these photosynthetic processes during low temperatures, as well as post-transcriptional activation, increase the expression of sucrose synthesis enzymes, alter the expression of Calvin cycle enzymes, change the leaf protein content, and affect plant photosynthesis process. Plant hormones and their derivatives play a role in how plants react to cold temperatures (Fowler and Thomashow, 2002). Several hormones such as abscisic acid (ABA) and cytokinin (CA), brassinosteroids (BRs), gibberellins (GBs), salicylic acid (SA), and jasmonic acid (JA) contribute significantly under low-temperature stress (Ashraf and Rahman, 2018). Low-temperature stress causes plants to release hormones. Many studies suggest that plant hormones affect low-temperature responsiveness. Under low-temperature stress, plant response genes were ABA-dependent and ABA-independent, depending on ABA/AREB and DREB/CBF. The intracellular auxin response regulates cold-stressed plant growth. Salicylic acid reduces stress damage by boosting proline, antioxidants, heat shock protein, secondary metabolism, and sugar, improving plant stress tolerance. Low-temperature stress increases the endogenous salicylic acid level in cucumbers and the expression of COR genes in grafted cucumbers. Hormones such as ethylene boost cold resistance by enhancing the antioxidant enzyme system’s activity. Under low-temperature stress, CRF2 and CRF3 regulate lateral root growth in *A. thaliana*. In fact, under low temperatures, hormone response is coordinated or antagonistic in horticultural plants. Thus, the plant hormone system’s dynamic balance network is developed, which helps plants maintain normal growth and development under stress. The mitogen-activated protein kinase cascade is a signaling transduction module that converts extracellular inputs into intracellular responses. Many mitogen-activated protein kinases (MAPKs) have been found in horticultural plants, including tomatoes and apples, based on whole-genome sequencing and transcriptome analysis. Recent research has shown that the MAPK cascade is also important in the biotic and abiotic stress responses of horticulture plants, including low temperatures. The genes and transcription factors are triggered in plant hormone signal transduction and mitogen-activated protein kinase signaling pathways (Wu, 2022) to enhance plant immunity (Li et al., 2019) and increase tolerance. Mitogen-activated protein kinases are required to integrate diverse intracellular signals transmitted by stress-induced secondary messengers. Recent research indicates that mitogen-activated protein kinase signaling regulates the cold stress response in *A. thaliana* (Zhao et al., 2017).

Plants need to be shielded from the cold’s destructive effects. Damage from chilling and freezing temperatures mimic that seen in natural settings. Greenhouses and tunnels provide significant protection from the cold for plants that are sensitive to the cold, but only if they are in good working order. Temperature fluctuations are less likely to cause damage in polyethylene-covered hoop homes. This is helpful for horticultural plants in preventing rapid temperature fluctuation within the greenhouse or tunnels. Perhaps a fast and exclusive strategy is needed to protect plants from cold temperatures in winter, and shading is one of the most fundamental and well-established techniques for crops implemented by growers all over the world (Mohotti, 2004). Shading is an effective strategy for limiting the quantity of sunlight that reaches tea plants. It helps to adjust important quality-related metabolites in tea leaves, improving tea growth, development, and flavor (Ku et al., 2010). Tea is a shade plant. In the past, we had successfully used different shading nets to protect tea plants from cold weather on physiological and physiochemical levels (Zaman et al., 2022), and shade is also one of the creative ways to protect plants from environmental factors (e.g., cold, chilling, frost, hot, light, and precipitation) and other harmful stresses or threats (Jiang et al., 2020) and cold stress (Zaman et al., 2022). The utilization of shade nets is a typical practice across various climatic settings to protect different crops on physiological, molecular, and cellular levels (Ji et al., 2018). Several researchers published studies on different tea cultivars in response to protective shading mechanisms under different environmental conditions (Ji et al., 2018; Zhang et al., 2022). Zaman et al. (2022) stated that different shade nets increased the physiological and biochemical attributes of *C. sinensis* (Zaman et al., 2022). Recently, a few studies were published on plant response to the shading on gene and transcriptional expression levels (Wu et al., 2020; Fang et al., 2022). The study of Wu et al. (2022) revealed that under three shading conditions compared to no-shade control plants, the phenotypic, physiological, and photosynthetic DEGs were enhanced in *Sambucus canadensis* (Wu et al., 2020). The authors also observed that the DEGs linked with photosynthesis, secondary metabolites, and other hormones were affected in shading groups compared to no-shade control plants. Thus, the result indicated an adaptive approach to retaining fitness under harsh conditions.

There are few studies on physiological responses, including DEG expression to cold stress in tea plants (Hao et al., 2018a; Hao et al., 2018b). Early low temperatures (4°C–16°C) may improve the tea plant’s cold acclimation, and enzyme activities increase after 4°C–5°C of acclimatization (Li et al., 2019). Low temperatures affect several leaf metabolites, including tea plants, in which small RNAs changed after 1–48 h at 4°C (Zhang et al., 2014). These investigations are bound to one or a few varieties within specific environments and cannot find global responses across natural environments and germplasm variability. Consequently, our understanding of the dynamic changes of DEG expression and the transcription factors and regulatory mechanisms of *C. sinensis* under different shading nets is still limited. Smart, short-term, and resilient agrarian solutions are essential to alleviate the harmful effects of critical environmental stresses and enhance the growth and productivity of tea plants under low temperatures. Therefore, in the present study, we used high-throughput RNA-seq of tea cultivar *C. sinensis* cv. Zhong Cha 102 to identify the important DEG expression and transcription factors responsible for enhancing cold-tolerance mechanism with response to shading during low temperatures. Several DEGs and transcription factors enriched in the present study play an essential role in photosynthesis, plant hormone signaling, and mitogen-activated protein kinase signaling transduction in three shading groups compared with no-shade control plants in November and December during low temperatures.

The aim of this study is to understand the physiological and transcriptome responses of important DEGs and transcription factors regulated under shading and no-shade responsible for the cold-tolerance mechanism of *C. sinensis* during low temperatures.

## 2 Materials and methods

### 2.1 Plant material and experimental setup

Experiments were carried out in the experimental field throughout during low temperatures at Rizhao, Shandong, China (35°514′N, 119°662′E). In this experiment, we tested the 5-year-old Zhong Cha 102 C*. sinensis* cultivar in three shading groups compared to the no-shade group during low temperatures. Above the tea leaves, a single layer of black polytene shades was set up in four rows, each 50 m long and supported by four metal fence posts. The main difference between black clothing shades was their intensity, such as 0% no shade or control (SD1), 30% of shading (SD2), 60% of shading (SD3), and 75% shading (SD4) were used (**Table 1**). The trial was carried out in November and December 2021, and the first harvesting was performed at 11:00 a.m. (24/11) and the second at 11:00 a.m. (23/12) during day time. The first, second, and third upper mature leaves were collected from each replication. A total of 30 sub-samples were collected in three shading groups compared to the no-shade control. All samples with six biological replicates were obtained from each row, carefully stored with high moisture in light barrier laminate packaging, and sent to the laboratory for further physiological and gene expression analyses.

**Table 1:** Shade specification, measurement, and PAR rate under three shading treatments compared to non-shade control plants in November and December during low temperature.

			November	December
Treatments	Shade cloth specification	Shade level (%)	PAR (μmol m−2 s−1)	PAR (μmol m−2 s−1)
SD 0	None	0%	793 a	530 a
SD 1	Black polyethylene net curtains	30%	353 b	262 b
SD 2	Black polyethylene net curtains	60%	261 c	202 c
SD 3	Black polyethylenenet curtains	75%	77 d	48 d

Specification as per the manufacturer: Treatments; SD0 as control, SD1 30% shading, SD2 60% shading, and SD3 75% shading. Black polyethylene net curtains, single layer, purchased from Shouguang lvyuan plastic products factory (Weifang, China). The photosynthetically active radiation (PAR). Different letters represent a significant difference between the treatments indicated by LSD test at p < 0.05.

### 2.2 Physiological attributes

#### 2.2.1 Cold damage analysis

Cold plant injuries in three shading groups and no-shade control plants were recorded. Browning symptoms (on leaf tissue) were confirmed to be a cold-damage indication (Alisoltani et al., 2015), and the percentage of leaf incurring cold injury on each plant was noted according to Zhang et al. (2020) and Zaman et al. (2022).

#### 2.2.2 Chlorophyll fluorescence measurement

A portable photosynthesis system (Li-6400XT, LI-COR, Inc., Lincoln, NE, USA) was used to measure chlorophyll fluorescence. The photochemical efficiency of photosystem II (Fv/Fm) was also measured. In brief, the fourth developed leaf from the shoot tip was acclimated in the dark for 30 min in three shading groups compared with no-shade control plants during low temperatures. As mentioned in our previous work, soil plant analysis development (SPAD) values in three shading groups and no-shade control plants were also recorded (Zaman et al., 2022).

#### 2.2.3 Determination of chlorophyll and carotenoid contents

For measuring the chlorophyll and carotenoid concentrations in three shading groups in comparison with no-shade control plants during low temperatures, 50 g of fresh tea leaves was cut into small pieces and kept in the dark at 25°C for 48 h in acetone–anhydrous ethanol solution (1:1) with minor modifications, and data were recorded according to our previous work (Zaman et al., 2022).

### 2.3 Transcriptome analysis

Tea leaf total RNA was extracted using RNAprep Pure, and the library was constructed following the RNA detection method. After quality control, 150-bp paired-end reads were generated using Illumina HiSeq. HISAT2 software utilized the aligned reference genome of *C. sinensis* ‘Shuchazao’. Feature Counts program tallied the genes (Kim et al., 2015). FPKM quantified gene expression mapped reads will be released on 14 February 2023 in the database of the National Center for Biotechnology Information (NCBI) with accession number PRJNA905739. DESeq2 software examined DEGs between comparison groups (Liao et al., 2014). The Benjamini–Hochberg method was used to correct *p*-values and calculate the false discovery rate (FDR). |log2 (fold change)|2 or FDR = 0.05 was used to screen DEGs. After adjustment, Kyoto Encyclopedia of Genes and Genomes (KEGG) pathways with *p* = 0.05 were considered enriched. Transcriptome analysis was completed at Wuhan Metware Biotechnology Co., Ltd.

### 2.4 qRT-PCR analysis

To verify the accuracy of the transcriptome data, 11 DEGs were selected for expression-level validation. Primers were designed using Beacon Designer 8, and the primer sequences are shown in **Supplementary Table 6**. Quantitative real-time PCR (qRT-PCR) was performed on an analytikjena-qTOWER2.2 fluorescence quantitative PCR instrument (Germany) using 2× SYBR^®^ Green master mix (DF, China). Three biological replicates were analyzed, with glyceraldehyde 3-phosphate dehydrogenase (CsGAPDH) gene used as the internal reference gene.

### 2.5 Statistical analyses

Statistix 8.1 software was used to determine significant differences between groups, and one-way analysis of variance (ANOVA) and Duncan’s multiple-range tests (*p*-values < 0.05) were used to analyze physiological and gene expression data under shading group and no-shade control plants during low temperatures. GraphPad Prism software was used for making figures. Graphics were formatted using Adobe Photoshop CC 2019.

## 3 Results

### 3.1 Weather situation under shading during low temperatures

**Figure 1:**
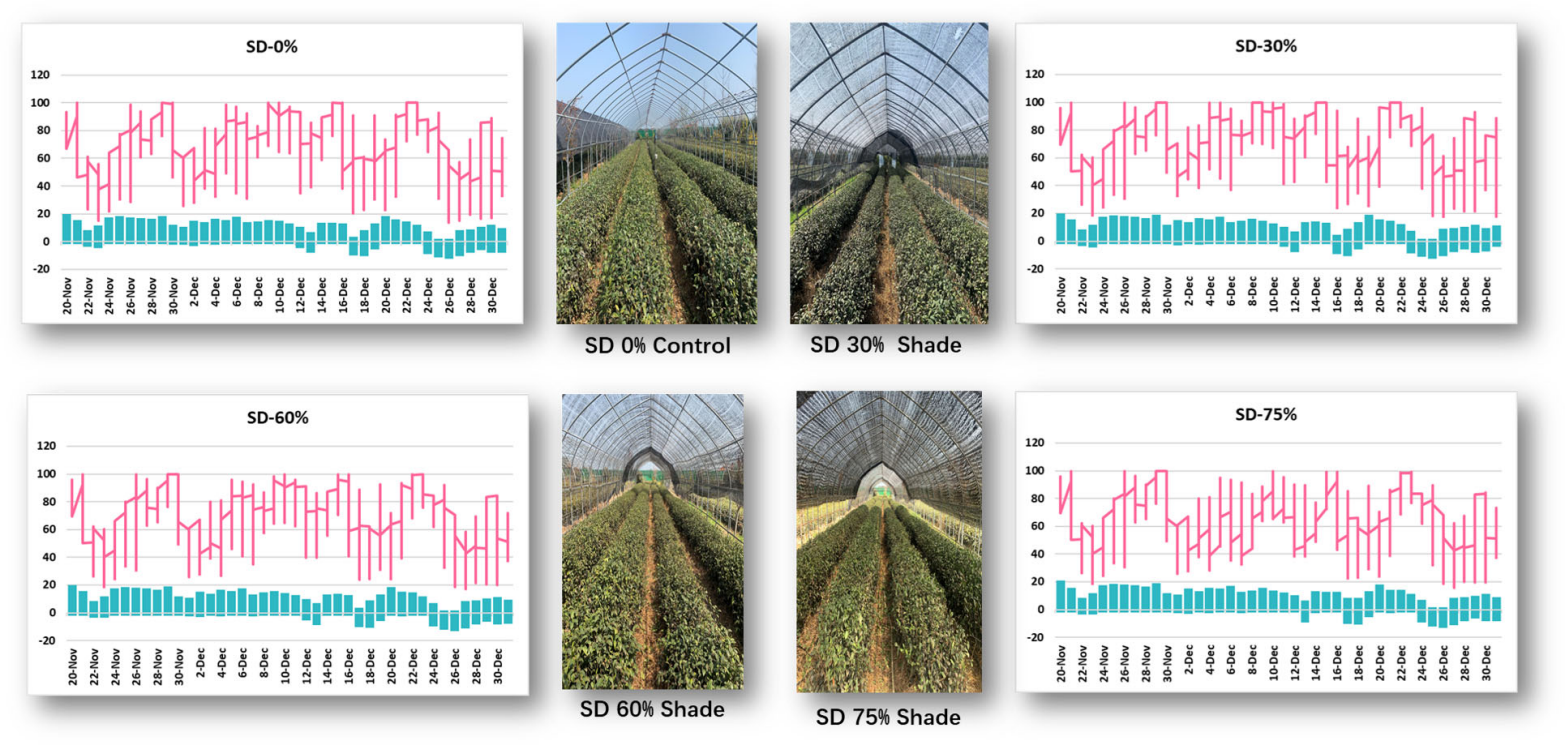
Weather situation under shading and no-shade conditions in November and December during low temperatures.

Monthly air temperature and relative humidity were measured at Rizhao, Shandong, China, from November to December 2021 in three shading groups compared to no-shade control plants during low temperatures (**Figure 1**). In November, the maximum air temperature was 18°C, the minimum temperature was −2.6°C, the maximum RH was 100%, and the minimum RH was 15.1%. Under SD0% control, the highest temperature was 16.4°C, the minimum temperature was −10.5°C, the maximum RH was 100%, and the minimum RH was 13.4%, as recorded in December 2021. Similarly, under SD30%, the maximum temperature was 17.9°C, the minimum temperature was −2.4°C, the maximum RH was 100%, and the minimum RH was 18.9%, as recorded in November; the maximum temperature was 16.9°C, the minimum temperature was −10.6°C, the maximum RH was 100%, and the minimum RH was 17.4% in December. In November, the maximum temperature was 18.1°C, the minimum temperature was −1.9°C, the maximum RH was 100, and the minimum RH was 18.9%. In December, the maximum temperature was 16.6°C, the minimum temperature was −11.1°C, the maximum RH was 100%, and the minimum RH was 17.3%. Similarly, in November, the maximum temperature was 19.1°C, the minimum temperature was −1.9°C, the maximum RH was 100%, and the minimum RH was 18.9%; in December, the maximum temperature was 16.3°C, the minimum temperature was −11.2, the maximum RH was 99.2%, and the minimum RH was 15.7%, recorded under SD75% of shading during low temperatures. Throughout this investigation, the highest temperature was 19.1°C, the lowest temperature was −11.2°C, the highest relative humidity was 100%, and the lowest was 13.4% (**Supplementary Table 1**).

### 3.2 Physiological attributes of *Camellia sinensis* under shading during low temperatures

**Figure 2:**
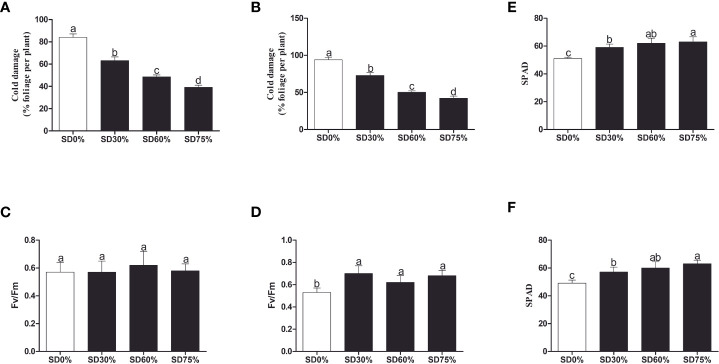
Tea plant performance under shading and no-shade in November and December during low temperatures. **(A)** Cold damage % in November. **(B)** Cold damage % in December. **(C)** Photochemical efficiency (Fv/Fm) in November. **(D)** Photochemical efficiency (Fv/Fm) in December. **(E)** Soil plant analysis development (SPAD) in November. **(F)** Soil plant analysis development (SPAD) in December. Different letters on the top of the columns within each figure represent significant differences between the treatments indicated by LSD test at *p* < 0.05. LSD, least significant difference.

To investigate the effect of shading on *C. sinensis*, the maximum cold injury was found under SD0% no-shade conditions throughout the experiment. The three levels of shading treatment significantly reduced plant cold damage compared to no-shade control plants under low temperatures. Interestingly, SD75% of shading-treated plants had the lowest cold injury in November and December. However, leaf injury was more severe in December than in November for all the treatments due to low temperatures (**Figures 2A, B**). Light quality has a significant influence on photosynthesis in plants. Photochemical efficiency (Fv/Fm) is the light quality that significantly impacts plants’ photosynthesis. Fv/Fm illustrates the efficiency of light energy conversion inside the PSII reaction center or plants’ possible maximum photosynthetic capability. As demonstrated in **Figures 2C, D**, the Fv/Fm efficiency was not significantly different in the three shading groups compared to no-shade control plants during low temperatures. However, a substantial decrease in Fv/Fm was seen in the SD0% no-shade control plants compared to the three shading groups in December during low temperatures in winter. The SPAD value is a parameter that evaluates the plant’s relative photosynthetic pigments or indicates the plant’s degree of greenness. As shown in **Figure 2E**, the SPAD value under unshaded control plants SD0% (50.700) was significantly lower than that under SD30% (58.700) and SD60% of shading (62.120 and 63.160) and significantly increased in SD75% of shading in November. Similarly, the minimum value (49.260) under SD0% (56.620), SD30% (60.080), and SD60% of shading (63.200) was significantly recorded under SD75% of shading in December during low temperatures (**Figure 2F**).

**Figure 3:**
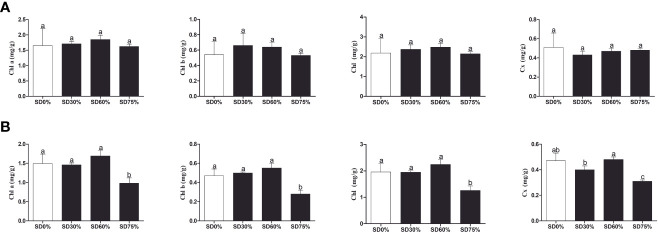
Physiological attributes of tea plants under shading and no shade in November and December during low temperatures. **(A)** Chlorophyll (Chl *a*), chlorophyll (Chl *b*), chlorophyll (Chl), and carotenoid (Cx) contents in November. **(B)** Chlorophyll (Chl *a*), chlorophyll (Chl *b*), chlorophyll (Chl), and carotenoid (Cx) contents in December. Different letters on the top of the columns within each figure represent significant differences between the treatments indicated by LSD test at *p* < 0.05. LSD, least significant difference.

As shown in **Figure 3A**, the chlorophyll *a* and *b*, chlorophyll, and carotenoid levels were not significantly different in the three shading groups compared to no-shade control plants during low temperatures in November. In the shading group, chlorophyll *a* and *b* and total chlorophyll (Chll) slightly increased as compared to no-shade control plants. The carotenoid (Cx) content increased in the no-shade control plants compared to the three shading groups. Similarly, chlorophyll *a* and *b* and chlorophyll (Chll) increased in the shading group, but there were no significant differences between SD30%, SD60%, and no-shade control plants during November. However, under SD75% of shading, the levels of chlorophyll *a* and *b* and chlorophyll (Chll) and carotenoid (Cx) were significantly reduced in comparison with no-shade control plants in December during winters (**Figure 3B**). These indicate that the cold temperature in December affects tea leaves’ green pigmentation during low temperatures.

### 3.3 Transcriptome profiles of *Camellia sinensis* under shading during low temperatures

Three shading groups were compared with no-shade control plants during low temperatures to understand the effects of shading on the molecular mechanisms of *C. sinensis*. The tea leaf samples in three shading groups were compared with no-shade control plants used for RNA-seq analysis with three biological replicates. A total of 165.3 Gb of clean data was obtained from 24 libraries. Each library was corrected with a Q20 percentage greater than 96.80%, and Q30 is 91.38%. The quality of each sample in transcriptome data was high and suggested that the analysis of transcriptome data was accurate and reliable (**Supplementary Table 2**).

### 3.4 Comparison of DEGs under shading during low temperatures

**Figure 4:**
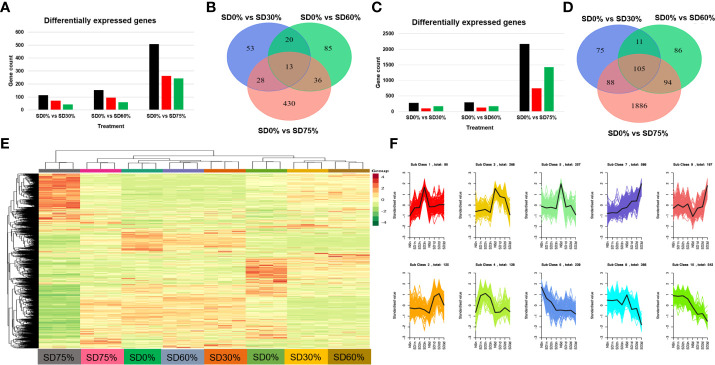
Transcriptome analysis of tea plant under shading and no shade in November and December during low temperatures. **(A)** Number of differentially expressed genes under SD0% *vs.* SD30%, SD0% *vs.* SD60%, and SD0% *vs.* SD 75% during November **(B)** Venn diagram of differentially expressed genes (DEGs) and shared DEGs under SD0% *vs.* SD30%, SD0% *vs.* SD60%, and SD0% *vs.* SD 75% during November. **(C)** Number of differentially expressed genes under SD0% *vs.* SD30%, SD0% *vs.* SD60%, and SD0% *vs.* SD 75% during December. **(D)** Venn diagram of differentially expressed genes and shared DEGs under SD0% *vs.* SD30%, SD0% *vs.* SD60%, and SD0% *vs.* SD 75% during December. **(E)** Heatmap of DEG regulation under different shading and no-shade control plants in November and December. **(F)** Cluster analysis of differentially expressed genes under different shading and no-shade control plants in November and December.

In the present study, we conducted an inclusive transcriptome analysis using RNA-seq of tea leaves in three shading groups compared to no-shade control plants during low temperatures. Our goal was to understand better the processes by which tea leaves were affected by low temperatures. We were able to obtain 20,807 DEGs (**Figure 4E**). The patterns of these DEGs are combined into 10 primary cluster categories (**Supplementary Table 3**). In comparison with no-shade control plants, sub-class 1 (99 genes), sub-class 2 (125 genes), sub-class 3 (268 genes), sub-class 4 (128 genes), sub-class 5 (257 genes), sub-class 6 (239 genes), sub-class 7 (586 genes), sub-class 8 (366 genes), sub-class 9 (197 genes), and sub-class 10 (542 genes) showed a different pattern of response in response to three shading groups in November and December under low temperatures (**Figure 4F**). We compared the DEGs of each shading group with no-shade control plants during low temperatures, for instance, SD0% *vs.* SD30%, SD0% *vs.* SD60%, and SD0% *vs.* SD75% of shading in November and December during low temperatures. In all group comparisons, a total of 3,523 DEGs were found. **Figures 4A, B** show that the number of downregulated DEGs was higher than that of upregulated DEGs. This indicates that cold temperature affected the numbers of DEGs and transcription factors in three shading groups compared with no-shade control plants during low temperatures. In the Venn diagram, 13 DEGs were shared in three shading groups in comparison with no-shade control plants during low temperatures in November, and 105 DEGs were shared in December in three shading groups in comparison with no-shade control plants during low temperatures (**Figures 4C, D**).

### 3.5 GO functional enrichment analysis under shading during low temperatures

**Figure 5:**
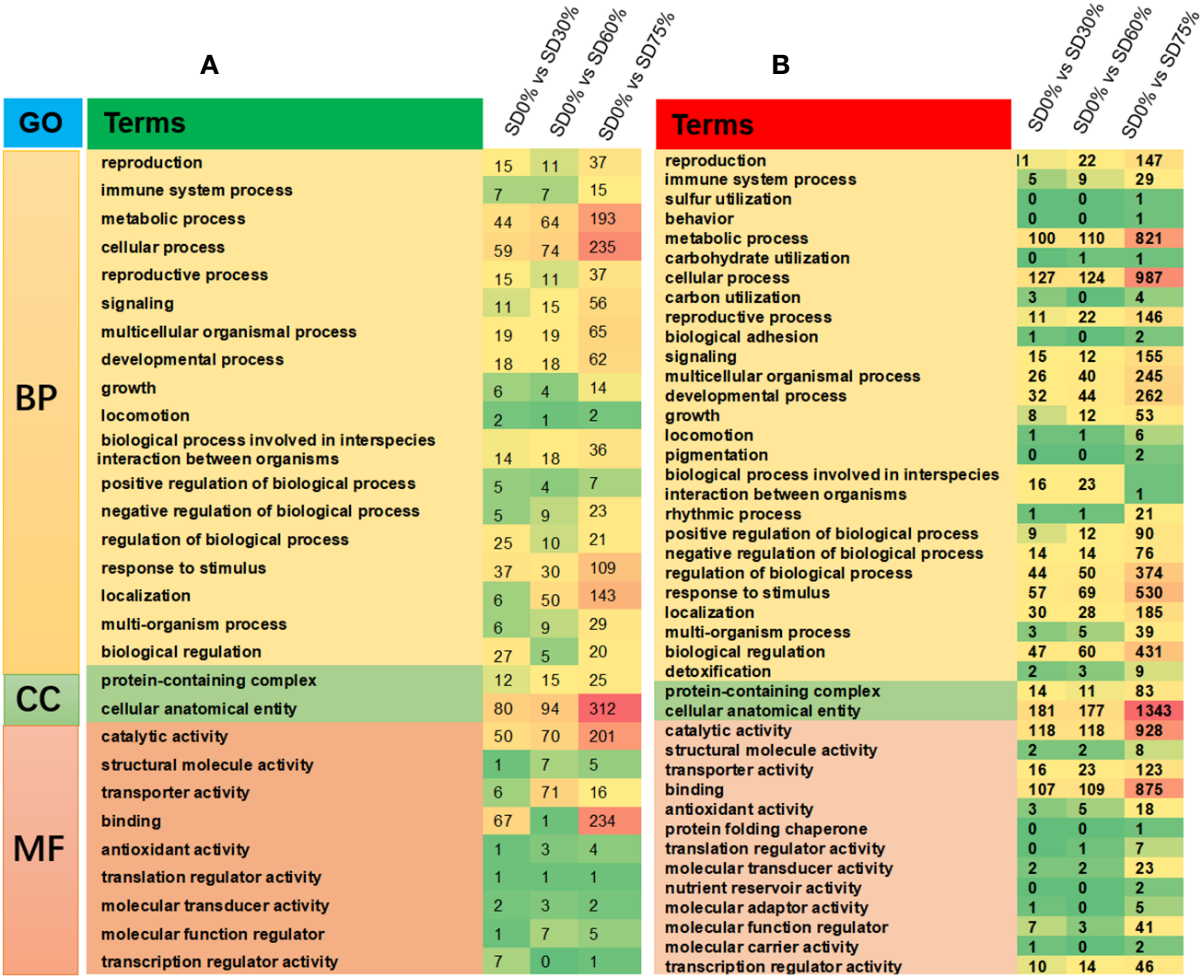
The classification of Gene Ontology (GO) terms of tea plants under shading and no shade in November and December during low temperatures. **(A)** Representation of DEGs enriched in biological process (BP), cellular component (CC), and molecular function (MF) under SD0% *vs.* 30%, SD0% *vs.* 60%, and SD0% *vs.* SD75% in November. **(B)** Representation of DEGs enriched in BP, CC, and MF under SD0% *vs.* 30%, SD0% *vs.* 60%, and SD0% *vs.* SD75% in December. DEGs, differentially expressed genes.

The DEGs were estimated using Gene Ontology (GO) and the KEGG pathway to identify the genes associated with tea leaves in three shading groups compared with no-shade control plants in November and December during low temperatures. These genes are mainly involved in cellular processes, metabolic processes, signaling, response to stimulus, growth, and regulation of biological processes in the biological process (BP) category. In the cellular component (CC) process category, the enriched genes were involved in protein-containing complex cellular, anatomical entities. Similarly, genes related to catalytic activity, binding, transcriptional regulator activity, molecular transducer activity, and molecular function regulator enriched in the molecular function (MF) category under SD0% *vs.* SD30%, SD0% *vs.* SD60%, and SD0% *vs.* SD75% in three shading groups were compared with no-shade control plants in November and December during low temperatures (**Figures 5A, B**; **Supplementary Table 4**).

### 3.6 Pathway enrichment analysis under shading during low temperatures

**Figure 6:**
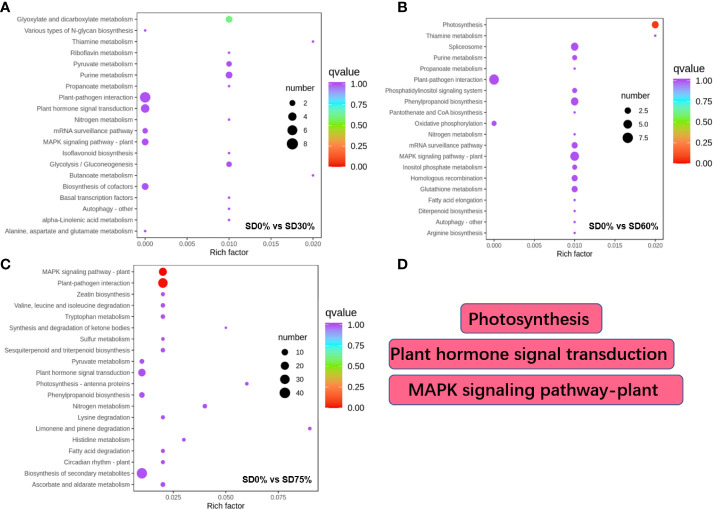
The numbers of DEGs expressed in Kyoto Encyclopedia of Genes and Genomes (KEGG) pathways in tea plants under shading and no shade in November and December during low temperatures. **(A)** Numbers of DEGs enriched under SD0% *vs.* SD30%. **(B)** Numbers of DEGs enriched under SD0% *vs.* SD60%. **(C)** Numbers of DEGs enriched under SD0% *vs.* SD75%. **(D)** The key pathways in present study were photosynthesis, plant hormone signal transduction, and MAPK signaling pathway. DEGs, differentially expressed genes.

Different DEGs were expressed into various KEGG pathways under shading and no-shade control plants in November and December during low temperatures (**Supplementary Table 5**). The key pathways focused on in this study were photosynthesis, plant hormone signal transduction, and mitogen-activated protein kinase signaling transduction plant (**Figure 6D**). Interestingly, plant hormone signal transduction and mitogen-activated protein kinase signal transduction were enriched under SD0% *vs.* SD30% (**Figure 6A**). Photosynthesis and mitogen-activated protein kinase signal transduction were enriched under SD0% *vs.* SD60% (**Figure 6B**), and photosynthesis-antenna, plant hormone signal transduction, and mitogen-activated protein kinase signal transduction were enriched under SD0% *vs.* SD75% during low temperatures (**Figure 6C**). The KEGG enrichment analysis showed that significant enrichment pathways increased with low temperatures and that the DEGs significantly enriched in these pathways in three shading groups compared with no-shade control plants are likely to play essential roles in coping with low-temperature stress in tea leaves (**Figure 6**).

### 3.7 Key DEGs in photosynthesis pathway under shading during low temperatures

**Figure 7:**
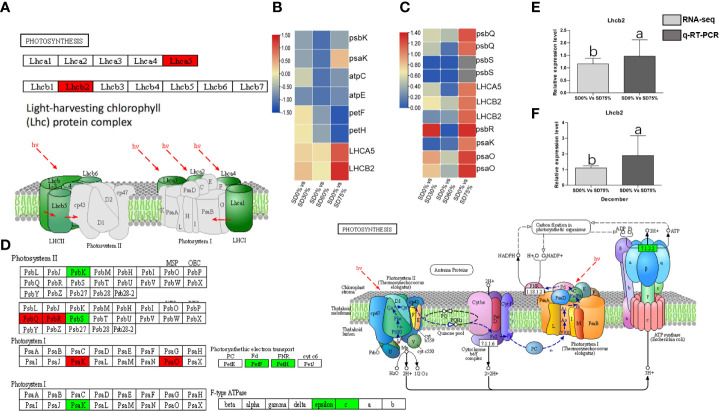
The numbers of DEGs were expressed in photosynthesis pathway in tea plants under shading and no shade in November and December during low temperatures. **(A)** Numbers of DEGs represented in red photosynthesis. **(B)** Heatmap representation of key DEGs’ regulation in photosynthesis pathway under SD0% *vs.* SD30%, SD0% *vs.* SD60%, and SD0% *vs.* SD75% in November during low temperatures. **(C)** Heatmap representation of key DEGs’ regulation in photosynthesis pathway under SD0% *vs.* SD30%, SD0% *vs.* SD60%, and SD0% *vs.* SD75% in December during low temperatures. **(D)** The numbers of DEGs were expressed in photosystem II and photosystem I; DEGs with green color indicate downregulation, and DEGs in red color indicate upregulations. **(E)** The relative expression level of key DEG Lhcb2 was validated by qRT-PCR under SD0% *vs.* SD75% in November. **(F)** The relative expression level of key DEG *Lhcb2* was validated by qRT-PCR under SD0% *vs.* SD75% in December. DEGs, differentially expressed genes.

The expression of crucial DEGs in the photosynthesis pathway led us further to evaluate the functional annotation of these expressed DEGs. We find that the main DEGs-encoded proteins were found in chloroplasts, where they act as antenna proteins, components of photosystems I and II, cytochromes, and electron transporters (**Figure 7**). A comparison of these DEGs’ expression in three shading groups in comparison with no-shade control plants in November and December during low temperatures revealed seven genes that had similar patterns of expression, and some of these DEGs were regulated more than one time. Interestingly, we find that light harvesting in photosystem I gene *Lhca5* and light harvesting in photosystem II gene *Lhcb2* had quite different expression patterns under SD0% *vs.* SD75% in November and December under low temperatures (**Figure 7C**). *Lhca5* encrypts a protein in the membrane involved in photosynthesis system I, and Lhcb2 encodes light-harvesting chlorophyll *a*/*b*-binding (LHC) proteins that constitute the antenna system of the photosynthetic apparatus (**Figure 7A**). We validated the key DEG *Lhcb2* under SD0% *vs.* SD75% in both November and December during low temperatures. The expression level using qRT-PCR is shown in **Figures 7E, F**. Hence, high expressions of *Lhcb2* were positively correlated in November and December during low temperatures. The other two essential genes *PsbQ* photosystem II subunit q and *PsbR* subunit of photosystem II were highly expressed under SD0% *vs.* SD75% in November under low temperatures (**Figures 7C, D**). At the same time, the subunit K of photosystem I reaction center *PsaQ* and subunit of photosystem I *PsaO* were highly expressed under SD0% *vs.* SD75% in December under low temperatures (**Figures 7C, D**). As mentioned earlier, all DEGs were highly expressed during low temperatures in November and December (**Supplementary Table 7**). Other essential DEGs of PSII K protein *psbK*, photosystem II subunit s *psbS*, subunit K of photosystem I reaction center *psaK*, two photosynthetic electron transporter, and multi-subunit enzyme energy-transducing membranes of chloroplasts were downregulated under SD0% *vs.* SD60% and SD0% *vs.* SD75% in three shading groups compared to no-shade control plants during low temperatures (**Figures 7B, D**).

### 3.8 Key DEGs and TFs activated in signal transduction pathways under shading during low temperatures

**Figure 8:**
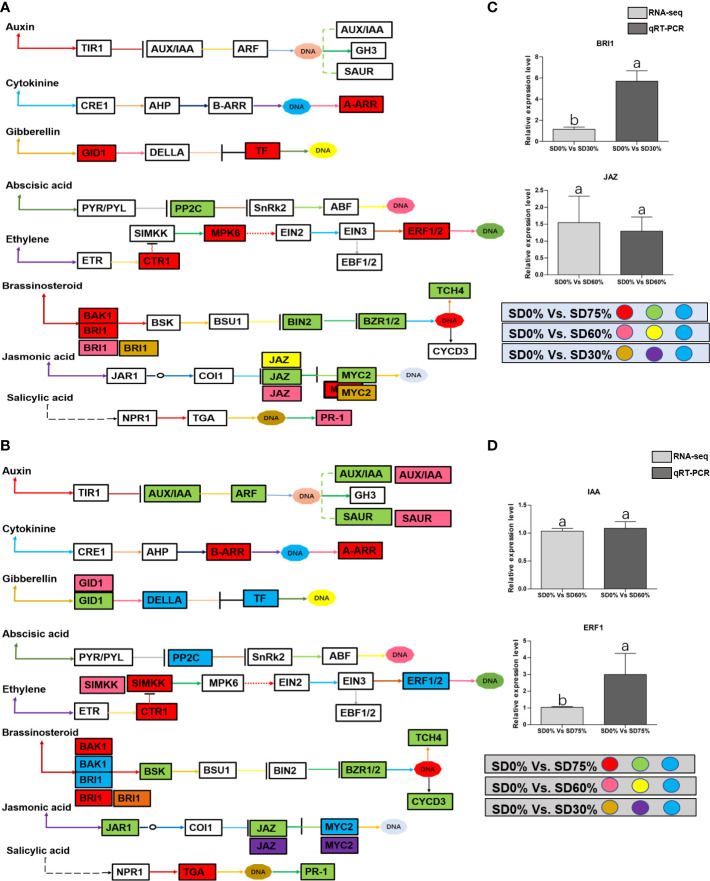
**(A)** The numbers of DEGs were expressed in Plant hormone signal transduction pathway in tea plant under shading and no-shade in November during low temperature. First, the up-regulation of these DEGs in red, down-regulation in green and both up and down regulation in sky blue color under SD0% Vs. SD75%. Second, the up-regulation of DEGs in pink, down-regulation in yellow and both up and down regulation shown in sky blue colors under SD0% Vs. SD60%. Third, the up-regulation of DEGs in brown, down-regulation in purple and both up-and down regulation in sky blue colors under SD0% Vs. SD75% **(C)** The relative expression level of key DEGs *BRI1* and *JAZ* were validated by q-RT-PCR under SD0% Vs. SD30, SD0% Vs. SD60% and %SD0% Vs. SD75% in November during low temperature. **(B)** The numbers of DEGs were expressed in Plant hormone signal transduction pathway in tea plant under shading and no-shade in December during low temperature. First, the up-regulation of these DEGs in red, down-regulation in green and both up and down regulation in sky blue color under SD0% Vs. SD75%. Second, the up-regulation of DEGs in pink, down-regulation in yellow and both up and down regulation shown in sky blue colors under SD0% Vs. SD60%. Third, the up-regulation of DEGs in brown, down-regulation in purple and both up-and down regulation in sky blue colors under SD0% Vs. SD75% **(D)** The relative expression level of key DEGs *IAA* and *ERF1* were validated by q-RT-PCR under SD0% Vs. SD30, SD0% Vs. SD60% and %SD0% Vs. SD75% in November during low temperature.

**Figure 9:**
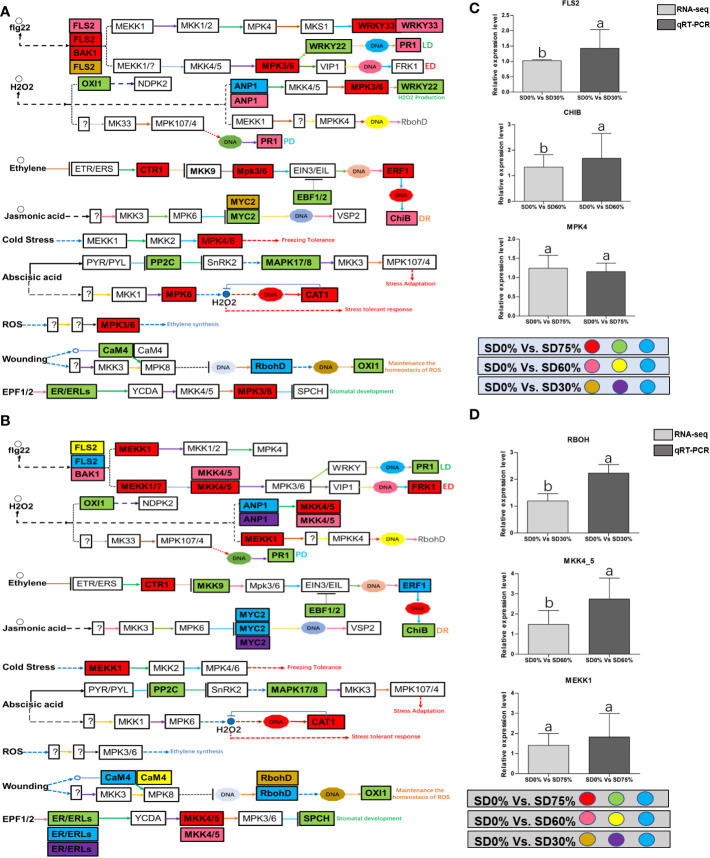
**(A)** The numbers of DEGs were expressed in MAPK signaling transduction pathway in tea plant under shading and no-shade in November during low temperature. First, the up-regulation of these DEGs in red, down-regulation in green and both up and down regulation in sky blue color under SD0% Vs. SD75%. Second, the up-regulation of DEGs in pink, down-regulation in yellow and both up and down regulation shown in sky blue colors under SD0% Vs. SD60%. Third, the up-regulation of DEGs in brown, down-regulation in purple and both up-and down regulation in sky blue colors under SD0% Vs. SD75% **(C)** The relative expression level of key DEGs *FLS2*, *CHIB* and *MPK4* were validated by q-RT-PCR under SD0% Vs. SD30, SD0% Vs. SD60% and %SD0% Vs. SD75% in November during low temperature. **(B)** The numbers of DEGs were expressed in *MAPK* signaling transduction pathway in tea plant under shading and no-shade in December during low temperature. First, the up-regulation of these DEGs in red, down-regulation in green and both up and down regulation in sky blue color under SD0% Vs. SD75%. Second, the up-regulation of DEGs in pink, down-regulation in yellow and both up and down regulation shown in sky blue colors under SD0% Vs. SD60%. Third, the up-regulation of DEGs in brown, down-regulation in purple and both up-and down regulation in sky blue colors under SD0% Vs. SD75% **(D)** The relative expression level of key DEGs *RBOH*, *MKK4_5* and *MEKK1* were validated by q-RT-PCR under SD0% Vs. SD30, SD0% Vs. SD60% and %SD0% Vs. SD75% in November during low temperature.

As most enrichment results are associated with signal transduction pathways, key DEGs and transcription factors generally play an essential part in these pathways, and we performed additional analysis on differentially expressed genes and transcription factors in three shading groups in comparison with no-shade control plants in November and December during low temperatures. The two signaling transduction pathways map (ko04075) “Plant hormone signal transduction” and map (ko04016) “mitogen-activated protein kinase signaling pathway” activated and enriched the key DEGs and transcription factors in three shading groups in comparison with no-shade control plants during low temperatures (**Supplementary Table 8**). Interestingly, 35 DEGs and transcription factors are associated with “Plant hormone signal transduction”. Four key DEGs and transcription factors were initially activated under SD0% *vs.* SD30%. SD0% *vs.* SD60% accounted for six key DEGs and transcription factors, and the late response of signaling triggered 25 key DEGs and transcription factors under SD0% *vs.* SD75%. However, 21 DEGs and transcription factors were shared in three shading groups compared with no-shade control plants in November and December during low temperatures, as summarized in **Table 2**. This indicates that key DEGs and transcription factors play an essential role during low temperatures in *C. sinensis*. These DEGs and transcription factors were mapped into hormone signal transduction of auxin (ABA), CA, abscisic acid (AB), GBs, ethylene (ET), BRs, JA, and SA, as shown in **Figure 8**. KEGG pathway enrichment analysis of DEGs and transcription factors revealed that the mitogen-activated protein kinase signaling pathway was the most prominent in the three shading groups compared with no-shade control plants in November and December during low temperatures. Furthermore, 39 DEGs and transcription factors are associated with the mitogen-activated protein kinase signaling pathway. Among them, three DEGs and transcription factors were enriched under SD0% *vs.* SD30%, nine DEGs and transcription factors were activated under SD0% *vs.* SD60%, and 27 DEGs and transcription factors were triggered under SD0% *vs.* SD75% of shading (**Table 3**). Twenty-two DEGs and transcription factors were found to be common in three shading groups compared to no-shade control plants in November and December during low temperatures. Most MAPK cascades were activated and transmitted as primary, intermediate, and late responses during cold temperatures. The expression of utmost key DEGs *MEKK1* and *MPK4/6* functioning in cold stress was significantly higher in November and December during low temperatures (**Figure 9**).

**Table 2:** DEGs involved in plant hormone signal transduction pathway.

	SD0% Vs. SD30%	
Gene Symbol	Manually annotated function	Shared DEG
SAUR	SAUR family protein	
BRI1	protein brassinosteroid insensitive 1	YES
JAZ	jasmonate ZIM domain-containing protein	YES
MYC2	transcription factor MYC2	YES
	SD0% Vs. SD60%	
IAA	auxin-responsive protein IAA	
GID1	gibberellin receptor GID1	
MKK4_5	mitogen-activated protein kinase kinase 4/5	
BAK1	brassinosteroid insensitive 1-associated receptor kinase 1	
BRI1	protein brassinosteroid insensitive 1	YES
MYC2	MYC2	YES
	SD0% Vs. SD75%	
IAA	auxin-responsive protein IAA	
ARF	auxin response factor	
ARR-B	two-component response regulator ARR-B family	YES
ARR-A	two-component response regulator ARR-A family	YES
GID1	gibberellin receptor GID1	YES
DELLA	DELLA protein	YES
PIF4	phytochrome-interacting factor 4	YES
PP2C	protein phosphatase 2C	YES
CTR1	serine/threonine-protein kinase CTR1	YES
MKK4_5	mitogen-activated protein kinase 4/5	
EBF1_2	EIN3-binding F-box protein	
ERF1	ethylene-responsive transcription factor 1	YES
BAK1	brassinosteroid insensitive 1-associated receptor kinase 1	YES
BRI1	protein brassinosteroid insensitive 1	YES
BSK	BR-signaling kinase	
BIN2	protein brassinosteroid insensitive 2	YES
BZR1_2	brassinosteroid resistant 1/2	YES
TCH4	Xyloglucan xyloglucans transferase TCH4	YES
CYCD3	cyclin D3, plant	
JAR1_4_6	jasmonic acid-amino synthetase	
JAZ	jasmonate ZIM domain-containing protein	YES
MYC2	transcription factor MYC2	YES
TGA	transcription factor TGA	
PR1	pathogenesis-related protein 1	YES
MPK6	mitogen-activated protein kinase 6	

**Table 3:** DEGs involved in MAPK signaling pathway.

	SD0% Vs. SD30%	
Gene Symbol	Manually annotated function	Shared DEG
ANP1	mitogen-activated protein kinase kinase ANP1	YES
MYC2	transcription factor MYC2	YES
ER/ERLs	LRR receptor-like serine/threonine-protein kinase ERECTA	
	SD0% VS SD60%	
WRKY33	WRKY transcription factor 33	
ANP1	mitogen-activated protein kinase ANP1	YES
PR1	pathogenesis-related protein 1	YES
CHIB	basic endochitinase B	YES
FLS2	LRR receptor-like serine/threonine-protein kinase FLS2	YES
BAK1	brassinosteroid insensitive 1-associated receptor kinase 1	YES
MKK4_5	mitogen-activated protein kinase 4/5	
CALM	Calmodulin	YES
ER/ERLs	LRR receptor-like serine/threonine-protein kinase ERECTA	YES
	SD0% VS SD75%	
WRKY33	WRKY transcription factor 33	
MPK3	mitogen-activated protein kinase 3	
WRKY22	WRKY transcription factor 22	
ANP1	mitogen-activated protein kinase ANP1	YES
CTR1	serine/threonine-protein kinase CTR1	YES
ERF1	ethylene-responsive transcription factor 1	YES
MPK4/6	mitogen-activated protein kinase 4/6	YES
MYC2	transcription factor MYC2	YES
PP2C	protein phosphatase 2C	YES
MAPK17_18	mitogen-activated protein kinase 17/18	YES
CALM	Calmodulin	YES
ER	LRR receptor-like serine/threonine-protein kinase ERECTA	YES
FLS2	LRR receptor-like serine/threonine-protein kinase FLS2	YES
BAK1	brassinosteroid insensitive 1-associated receptor kinase 1	YES
PR1	pathogenesis-related protein 1	YES
FRK1	senescence-induced receptor-like serine/threonine-protein kinase	
OXI1	serine/threonine-protein kinase OXI1	
MEKK1	mitogen-activated protein kinase 1	
PR1	pathogenesis-related protein 1	
MKK9	mitogen-activated protein kinase 9	
EBF1_2	EIN3-binding F-box protein	
MKK9	mitogen-activated protein kinase 9	
CHIB	basic endochitinase B	YES
CAT1	catalase	
RBOH	respiratory burst oxidase	
MKK4_5	mitogen-activated protein kinase 4/5	
SPCH	transcription factor SPEECHLESS	

## 4 Discussion

### 4.1 Physiological attributes of *Camellia sinensis* under shading during low temperatures

Temperature changes are among the most common abiotic stressors encountered by plants. Low-temperature stress affects plants’ various morphological, physiological, biochemical, and molecular characteristics, ultimately inhibiting plant growth and development (Wang et al., 2018). Therefore, short-term strategies are needed to alleviate important environmental stresses and increase plant growth and development. Shade is one of the finest strategies to protect plants from various temperature stressors (Zaman et al., 2022) to increase growth and productivity and reduce cold damage or leaf injury in plants (Zhang et al., 2022). The present study showed cold damage to *C. sinensis* leaves in three shading groups compared with no-shade control plants in November and December during low temperatures. The results demonstrate that shaded tea leaves were shielded from leaf injury in November and December during low temperatures compared to no-shade control plants. It means that shading protected tea leaves from cold damage due to stopping ice accumulation in extracellular tissues (Zuther et al., 2018). Therefore, it might be helpful for alleviating the harmful effects of cold temperatures. However, different shading conditions modify the intensity and affect the photosynthetic capacity of plant growth and development under different environmental conditions, thereby regulating plant tolerance to high photosynthetically active radiation (PAR) or low PAR (Rezai et al., 2018). Our study revealed that the PAR (μmol m^−2^ s^−1^) rate was significantly lower in three shading groups compared to no-shade control plants in November and December during low temperatures. Our results are like those of the previous study, which reported a higher PAR rate in the controlled group than in the shading groups (Thakur et al., 2019). We also measured the soil plant and development (SPAD) in three shading groups compared with no-shade control plants during low temperatures to quantify the relative amount of chlorophyll in the leaves. We found that SPAD values were significantly higher in three shading groups in comparison with no-shade control plants in November and December during low temperatures. Our results are consistent with those of previous studies (Kumari et al., 2014; Zaman et al., 2022). Increased SPAD values suggest higher chlorophyll concentrations in the leaf because plants grown under shade exhibited higher photosynthetic activity due to their higher photosynthetic pigments. Variations in photosynthetic pigmentation in response to low temperatures have received much attention in recent years. Photochemical efficiency is a reliable predictor for plant adoption under abiotic stress (Zuther et al., 2018), including low temperatures. However, we did not find many variations in the three shading groups compared to no-shade control plants in November during low temperatures. Nevertheless, when the temperature was colder in December, the cold weather affects the photochemical efficiency under no-shade control plants. These results indicate that cold reduces the photochemical efficiency, but different shades alleviated the harmful effects of low temperatures and sustained photochemical machinery, which might be the reason that shading is one of the effective systems in preventing leaf cold injury of tea plants during low temperatures. In fact, plants regularly interact with shade by strengthening light use efficiency through a shade tolerance strategy (Gong et al., 2015). In this work, we found no significant differences in chlorophyll *a* and *b*, chlorophyll, and carotenoid contents in November. A lower amount of chlorophyll *a* and *b* and a higher chlorophyll concentration were beneficial for photosynthesis under different shades, consistent with an earlier study (Li et al., 2020). The degradation in chlorophyll concentration under SD0% no-shade plants in November indicates that cold affects tea leaves and disrupts membrane fluidity in cell compartments during low temperatures. However, when the temperature decreases, we found that the concentrations of chlorophyll *a* and *b*, chlorophyll, and carotenoid were degraded under SD75% of shading in December during low temperatures. The reasons are unknown. Moving forward, under SD30% and SD60% of shade conditions, the concentrations of chlorophyll *a* and *b* and chlorophyll were higher as compared to no-shade control plants in December during low temperatures. This indicates that under different shade groups, the higher chlorophyll concentration and more pigment-binding proteins are associated with their photons collected by photosystem II per unit. The light-harvesting complex of photosystem II is mainly composed of chlorophyll *b* (Wu, 2022). In contrast, the low PAR, photochemical efficiency, and lower chlorophyll concentration in no-shade control plants indicate that cold temperatures might disrupt the membrane permeability in the cell compartment of tea leaves exposed to low temperatures. Thus, the above findings revealed that different shade groups protected against cold damage, improved the physiological attributes of tea leaves, and alleviated the harmful effects of cold during low temperatures.

### 4.2 Key pathways enriched in *Camellia sinensis* under shading during low temperatures

According to various transcriptome data, several genes and transcription factors play an essential role in photosynthesis, plant hormone signal transduction, and mitogen-activated protein kinase signal cascades under different abiotic stresses (Li et al., 2019; Ermilova, 2020; Ritonga and Chen, 2020; Aslam et al., 2022). In this study, the annotation of Gene Ontology and KEGG enrichment analysis demonstrated that low temperatures regulated the expression of several DEGs in the photosynthetic pathway of photosynthesis (Wu et al., 2020) and activated numerous DEGs and transcription factors in signal transduction pathways (Wang et al., 2018; Wu, 2022) under shade and no-shade control plants in November and December, respectively.

### 4.3 Photosynthesis-related DEGs revealed in *Camellia sinensis* under shading during low temperatures

Photosynthesis is the essential process that drives the growth and development of a plant. In the photosynthesis process, photosystem I and II pigment–protein complexes are related *via* the cytochrome complex and the electron transport chain (Mamedov et al., 2015) from plastocyanin to Fd (Busch and Hippler, 2011). In this state, the reaction centers of the thylakoid membrane receive light energy that pigments have collected in the LHC proteins in photosystem I. However, a unique complex called photosystem II can absorb light and break down water (Shi et al., 2012a). Oxygen-evolving enhancer proteins, including *PsbB*, *PsbC*, *PsbR*, *PsbO*, *PsbP*, and *PsbQ*, are essential components of the oxygen-evolving complex (Silveira and Carvalho, 2016). Photosystem I contains the reaction center of subunits *PsaA* and *PsaB*; binding subunits *PsaG*, *PsaK*, *PsaH*, *PsaL*, *PsaO*, and *PsaP*; ferredoxin docking subunits *PsaD* and *PsaE*; plastocyanin docking subunits *PsaF* and *PsaN*; and *FeS* apoprotein (Ichino et al., 2014). In the current study, photosynthesis-related GO terms and KEGG pathways differed significantly between the three groups compared to no-shade control plants. Photosynthesis-related DEGs are enriched in photosynthesis, photosynthesis-antenna proteins, and carbon fixation in photosynthetic organisms’ pathways. Photosynthesis involves photosystem II, cytochrome b6/f complex, electron transport, and ATP synthase. Most DEGs were increased and enriched in three shading groups compared with no-shade control plants in November and December during low temperatures. The enrichment and high expression of light-harvesting chlorophyll (LHCI), *Lhca5* in photosystem under SD0% *vs.* SD75%, may be a key DEG for coping with cold stress in November and December during low temperatures. LHCII-related DEG *Lhcb2* was increased under low temperatures. This suggests that shade promoted the regulation of LHC-related genes under low temperatures and improved the photosystem machinery of tea leaves during low temperatures. Our findings are congruent with those of a previous study in which authors discovered that the high expression of light-harvesting chlorophyll Lhca2 in the photosystem is an important contributor to cold tolerance in tea leaves (Li et al., 2019). In addition, the core complex subunits of photosystem II *PsbR* and *PsbQ* were highly expressed under SD0% *vs.* SD75% in December during low temperatures, and the photosystem I core complex contains reaction center subunits *PsaK* and *PsaO*, which were also highly expressed under SD0% *vs.* SD75% during low temperatures. At the same time, *psaK* was found to be downregulated under SD0% *vs.* SD75% in November during low temperatures. We validated the highly regulated key DEG *Lhcb2* expressed in November and December under shading and no-shade control plants, respectively. The relative expression level was satisfactory between RNA-seq and qRT-PCR analysis of *Lhcb2*.

### 4.4 Plant hormone signal transduction DEGs and TFs activated in *Camellia sinensis* under shading during low temperatures

In order to survive in low-temperature environments, plants have evolved a set of complex signaling pathways (Wang et al., 2018). Among them, plant hormone signal transduction is a crucial pathway for activating DEGs and transcription factors under different shading conditions (Lorrain et al., 2008; Gommers et al., 2017). These DEGs and transcription factors are active during low temperatures (Wu, 2022). In the present study, several critical DEGs and transcription factors were regulated in three shading groups compared with no-shade control plants in November and December during low temperatures. *BRI1* and *MYC2* were upregulated and *JAZ* was downregulated under SD0% *vs.* SD30%, and under SD0% *vs.* SD60%, *BRI1*, *JAZ*, and *PR-1* were expressed highly. *AA-R*, *GIDI1*, *CTRI1*, *MPK6*, *ERF1/2*, *BAK1*, and *BRI1* were also expressed highly and *PP2C*, *BIN2*, *BZR1/2*, *TCH4*, *JAZ*, and *MYC2* were downregulated in November during low temperatures. In contrast, *BRI1* was upregulated and *JAZ* and *MYC2* were downregulated under SD0% *vs.* SD30%. Under SD0% *vs.* SD60%, *AUX/AA*, *GIDI*, *SIMKK*, *BAK1*, *BRI1*, and *MYC2* were found to be expressed highly. However, *AUX/AA*, *ARF*, *SAUR*, *B-AAR*, *A-AAR*, *GIDI*, *DELLA*, *PPC2*, *CTRI*, *SIMKK*, *EBF1/2*, *ERF1/2*, *BAK1*, *BRI1*, *BSK*, *BZR1/2*, *TCH4*, *CYCD3*, *JAR1*, *JAZ*, *MYC2*, *TGA*, and *PR-1* were differentially regulated in December during low temperatures. This indicates that several important DEGs and transcription factors are associated with hormone signal transduction playing an essential role in three shading groups in comparison with no-shade control plants in November and December during low temperatures in *C. sinensis*, and the molecular mechanism of these DEGs is illustrated in **Figure 8**. Abiotic stress responses in plants are primarily regulated by the ABA signaling pathway, which causes profound variations in gene expression and adaptive physiological responses (Danquah et al., 2014). DEGs involved in the production of plant hormones like auxin (IAA), ABA and CA, BRs, GBs, SA, and JA are differentially expressed due to variations in the expression of genes triggered in the signal cascade mechanism (Wang et al., 2018). The signaling route is triggered by auxin in response to low temperatures. Auxin is the first hormone released and initiates the hormone signal transduction process in plants. Auxin/indole-3-acetic acid and *AUX/IAA* family, response factor *ARF* family, and the short auxin RNA-SAUR family (Luo et al., 2018) are all auxin-responsive factors that help plants swiftly detect and respond to changes in auxin levels under low-temperature stress. The results of this study demonstrated that DEGs of auxin play a significant part in mitigating the negative effects of cold weather by regulating the expression of *AUX1*, *ARF*, and *SAUR*. Moreover, most known reactions of plant cytokinin metabolism or signaling systems to low temperatures are inhibitory (Argueso et al., 2009), but the responses are complex (Pavlů et al., 2018). Interestingly, cold temporarily activates type A *ARR* expression in a cytokinin- and ethylene-dependent way (Shi et al., 2012b). In our work, cytokinin *B-ARR* and type A *ARR* were expressed highly in three shading groups compared with no-shade control plants in November and December during low temperatures. Our results demonstrate that these two cytokinins interact to manage cold stress signals and alleviate the harmful effects of low temperatures in tea leaves. These findings align with earlier research, in which the author stated that mutations in cytokinin-type genes increase cold tolerance in *A. thaliana* during cold temperatures (Jeon et al., 2010). The classic phytohormones that control plant growth and abiotic stressors are gibberellic acid. The soluble receptor of *GID1* controls plant growth and development by interacting with *DELLA* protein in the gibberellic signaling pathway. The involvement of these DEGs in signaling pathways improved cold tolerance, and the overexpression of *GID1* and *DELLA* was regulated during low temperatures in three shading groups compared with no-shade control plants during low temperatures. The enzymes involved in ABA synthesis are zeaxanthin epoxidase, 9-*cis*-epoxycarotenoid dioxygenase, and abscisic aldehyde oxidase (Finkelstein, 2013). In a previous study, two ZEP genes, *NCDE 1* gene and *SnRK*s, were identified and expressed differentially under cold stress (Li et al., 2019), which functioned as part of a double-negative regulatory network that enhanced ABA signal transduction with *PYR/PYL*, *PP2C*, and *SnRK2*. In our study, we found that *PPC2* regulated differentially in three shading groups in contrast with no-shade control plants in November and December during low temperatures might play an important role during cold stress. BRs are a class of steroid hormones that play an important role in plant growth and development (Sharif et al., 2022a). Both BRs and ET are well known for their roles in plant growth and development during biotic and abiotic stressors (Jiroutova et al., 2018), including low temperatures. Our research identifies DEGs and transcription factors involved in brassinosteroids and ethylene, which might induce cold tolerance in tea leaves in three shading groups in comparison with no-shade control plants in November and December during low temperatures, respectively; these DEGs and transcription factors may be cooperatively controlled to facilitate tea plant adaptation to cold stress. More studies are needed to fully understand how they interact to regulate when plants are subjected to cold conditions. Furthermore, plants use jasmonic acid and its derivative as signaling molecules in response to biotic or abiotic environmental stress. This activates a variety of jasmonic-related genes and transcription factors that control plants’ protective responses to various stresses (Gális et al., 2009). Among them, MYC family transcription factors are the important transcription factors involved in the hormone signaling transduction pathway in plants’ response to low temperatures. To put it simply, MYC2, one of the newly identified plants MYC transcription factors, which is found in the model plant *A. thaliana* and mostly used in in-depth investigations under different abiotic stress, plays a regulatory role by establishing the *COI1*/*JAZs*/*MYC2* complex (An et al., 2022). In this study, we also discovered DEGs of JAZ and transcription factor MYC2 expressed differentially in three shading groups in comparison with no-shade control plants in November and December during low temperatures. Our result is consistent with a previous study in which *MYC2*, *JAZ1*, *JAZ2*, *JAZ3*, and *JAZ12* are involved in the jasmonic signal transduction pathway and were significantly upregulated in *Phoebe bournei* under shade (An et al., 2022). We assume that the various shading strategies in our work are reliable for coping with cold tolerance in tea and played a significant part in the differential expression of JAZ and MYC2 during low temperatures. Moreover, **Figures 8A, B** show that there was a certain alteration in the expression of DEGs and transcription factors involved in the plant hormone signaling pathway, indicating a dramatic shift in hormone signaling. Undoubtedly, different stresses cause a cross-talk between hormone channels in plants (Murphy, 2015; Li et al., 2019). In fact, the supreme hormone auxin is still less understood in response to environmental stresses, which requires further exploration (Sharif et al., 2022b). Therefore, the reported findings of DEGs and transcription factors in our study are consistent with the hormonal cross-talk way that might raise cold tolerance and thereby alleviate cold damage in *C. sinensis* under low temperatures. However, we validated the high expression of key DEGs *BRI1* and *JAZ* under shading and no-shade control plants in November and *IAA* and *ERF1* in December. The relative expression level was reasonable between RNA-seq and qRT-PCR analysis of confirmed DEGs enriched in plant hormone signal transduction pathway (**Figures 8C, D**).

### 4.5 Mitogen-activated protein kinase cascades activated in *Camellia sinensis* under shading during low temperatures

**Figure 10:**
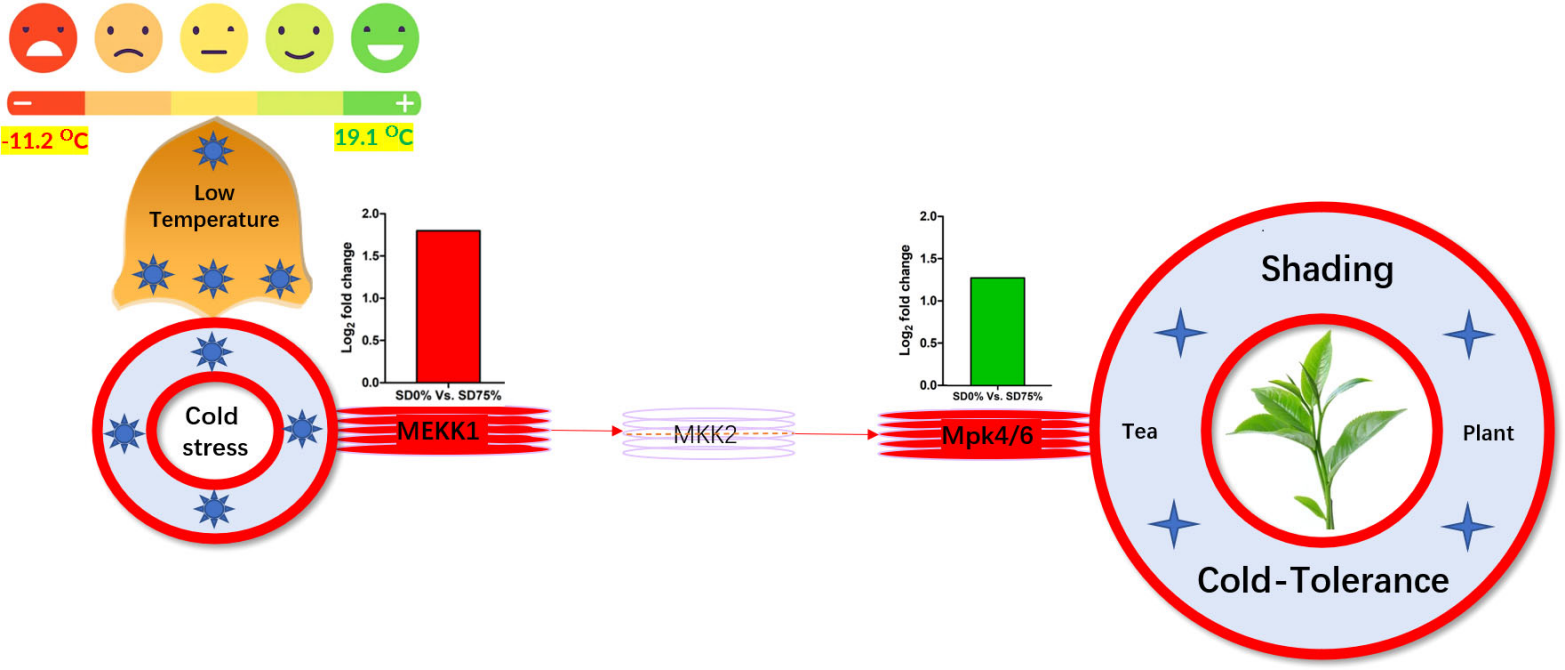
Systematic representation of key DEGs *MEKK1* and *MPK4/6* activated under shading in mitogen-activated protein kinase signaling pathway during low temperatures might be responsible for cold-tolerance mechanism of tea. DEGs, differentially expressed genes.

Mitogen-activated protein kinase cascades are essential in regulating many distinct biological processes, including cell function, proliferation, and response to various abiotic stresses (Hao et al., 2015), including low temperatures. In the current study, several WRKY, FLS, MYC2, ERLs, and ERFs and MAPKKK, MAPKK, and MAPK families were regulated with key DEGs that transmitted signaling in tea leaves in three shading groups in comparison with no-shade control plants in November and December during low temperatures. Though, the regulations of these genes and transcription factors transmitted signaling in three steps. In the first step, the initial cold sensor was triggered in cell compartments when tea leaves were subjected to less cold temperatures in three shading groups compared to no-shade control plants during low temperatures. However, when cold temperature increases, the high expression of DEGs triggered signal transduction into the nucleus in three shading groups compared with no-shade control plants during low temperatures. Moreover, the expression of upregulation and downregulation of DEGs and transcription factors in three shading groups in comparison with no-shade control plants was responsible for the transmission of signaling, and transduction events occurred inside the cell during low temperatures. This indicates that cold weather triggers the cascade, and weather fluctuations owing to different shades regulate the expression of DEGs and transcription factors in tea leaf cell compartments. The activation of these cascades may help maintain membrane permeability, preventing ice formation in cell compartments of tissues at low temperatures. Low temperatures restrict cell membrane fluidity and create ice formation in the cell compartment of plants. However, the cascade activation plays an essential role in signaling events’ genes and transcription factors, enhancing plant tolerance responses to low temperatures (Chen and Thelen, 2013). Regarding shading conditions, the GO categorization results of their DEGs are involved in cellular processes, membranes, signaling, and binding. According to the processes mentioned earlier, low-temperature signaling events occur in the cellular compartment and the transmission of signals cascades into the membrane and binds the membrane fluidity under low temperatures. The ethylene-responsive DEGs were highly expressed in three shading groups compared with no-shade control plants in November and December during low temperatures (**Figures 9A, B**). To cope with low temperatures, ET, ABA, and JA hormone derivatives might be transmitted signals under different shades. Our result is consistent with that of Müller and Munné-Bosch (2015), who reported that ET, ABA, and JA could activate ERF genes (Müller and Munné-Bosch, 2015). ERFs bind to the GCC box and DRE elements at low temperatures, providing cold tolerance to certain plants (Ritonga and Chen, 2020). Similarly, we identified the essential transcription factor families MAPKKK, MAPKK, and MAPK were activated in three shading groups compared with no-shade control plants in November and December during low temperatures. It indicates that mitogen-activated protein kinases play a crucial role in integrating diverse intracellular signals provided by shading-induced secondary messengers to low temperatures. In most cases, signal transduction will activate dormant MAPKKKs, the first kinase in the mitogen-activated protein kinases cascade. After being activated, MAPKKKs phosphorylate MAPKKs on a conserved serine/threonine and then trigger both (Ritonga and Chen, 2020). The signaling pathway of MAPK by activated MAPKKs is a further step in MAPK engagement. Mitogen-activated protein kinase signaling plays a role in *A. thaliana* cold stress response *via* regulating the ICE1 pathway (Zhao et al., 2017). Moreover, we also validated the highly expressed key DEGs regulated in the mitogen-activated protein kinase signaling transduction pathway. The key genes were *FLS2*, *CHIB*, and *MPK4* expressed in November and *RBOH*, *MKK4_5*, and *MEKK1* in December under shading and no-shade control plants, and the relative expression level was similar between RNA-seq and qRT-PCR analysis (**Figures 9C, D**). Moreover, we identified two significant DEGs, *MEKK1* and *MPK4/6*, enriched in the mitogen-activated protein kinase signaling pathway, a novel discovery for our tea cultivar Zong Cha 102. Both DEGs were highly regulated under shading groups compared with no-shade control plants in November and December during low temperatures. These DEGs are positive indicators for cold tolerance in *C. sinensis* (**Figure 10**). Our novel findings are consistent with Teige et al. (2004), in which the authors reported that *MEKK1* directly mediates *MPK4* and *MPK6* and that the regulation of these two key genes in the mitogen-activated protein kinase signaling pathway in model plant *A. thaliana* is mainly responsible for cold stress tolerance (Teige et al., 2004).

## 5 Conclusion

In summary, we revealed some novel physiological and transcriptomic information from the tea plant at low temperatures in three shading groups and no-shade control plants. We discovered that the adaptive regulation of tea leaves under shading conditions is primarily related to physiological characteristics such as photochemical machinery, photochemical efficiency of soil plant development, and chlorophyll content and gene expression analysis. In comparison to no-shade control plants, the shading group performed a main role in the protection mechanism of tea leaves against cold damage. Furthermore, DEGs and transcription factors associated with photosynthesis, plant hormone signaling, and mitogen-activated protein kinase signaling pathways played an important role in shading and no-shade control plants. The light-harvesting photosystem I gene *Lhca5* and light-harvesting photosystem II gene *Lhcb2* synthesized the CO_2_ fixation and maintained photosynthetic machinery, sustaining chlorophyll concentration. Finally, DEGs involved in plant hormone signaling and mitogen-activated protein kinase signaling cascades were activated, and shading might protect the tea plants from cold injury due by retaining the ice accumulation and maintaining membrane fluidity in cellular compartments of leaf tissues under low temperatures. Mitogen-activated protein kinase signal transduction DEGs *MEKK1* and *MPK4/6* are key players in this study for strengthening the cold-tolerance mechanisms in *C. sinensis*. Taking it all together, in this study, we confirm that different shading groups compared to no-shade conditions played a substantial role as a cover for sheltering tea cultivar Zhong Cha 102 on physiological and molecular levels during low temperatures. Also, shading is a fast and reliable technique for enhancing the cold-tolerance mechanism in *C. sinensis*. This study paves the way for researchers to better understand the protective approach for shielding horticultural crops under low-temperature scenarios.

## Data availability statement

The original contributions presented in the study are included in the article/**Supplementary Material**. Further inquiries can be directed to the corresponding author.

## Author contributions

Conceptualization of work and evaluation by SZ and ZD. Methodology and investigation by SW and DS. Physiological analysis performed by HW, XP, and MW. Physiological data analysis performed by JS, DS, and SD. Manipulation of transcriptome data and manuscript written by SZ. The article was revised and organized by IS. Project direction, resources, and funding achieved by YW and ZD. All authors contributed to the article and approved the submitted version.

## Supplementary material

SUPPLEMENTARY TABLE 1 Climatic data.

SUPPLEMENTARY TABLE 2 Summary of raw reads.

SUPPLEMENTARY TABLE 3 Cluster analysis.

SUPPLEMENTARY TABLE 4 GO terms.

SUPPLEMENTARY TABLE 5 Enrichment of KEGG pathways.

SUPPLEMENTARY TABLE 6 Primer’s information used in qRT-PCR detection.

SUPPLEMENTARY TABLE 7 DEGs regulations in photosynthesis pathway.

SUPPLEMENTARY TABLE 8 DEGs regulations in hormone signaling pathway and MPAK signaling transduction pathway.
